# A local *Talaromyces atroroseus* TRP-NRC isolate: isolation, genetic improvement, and biotechnological approach combined with LC/HRESI-MS characterization, skin safety, and wool fabric dyeing ability of the produced red pigment mixture

**DOI:** 10.1186/s43141-022-00335-2

**Published:** 2022-04-22

**Authors:** Rasha G. Salim, Mohamed Fadel, Yehya A. Youssef, Hanan A. A. Taie, Nivien A. Abosereh, Ghada M. El-Sayed, Mohamed Marzouk

**Affiliations:** 1 Microbial Genetic Department, Biotechnology Research Institute, National Research Centre, 33 El-Bohouth St. (Former El-Tahrir St.), Dokki, Cairo, 12622 Egypt; 2 Microbial Chemistry Department, Biotechnology Research Institute, National Research Centre, 33 El-Bohouth St. (Former El-Tahrir St.), Dokki, Cairo, 12622 Egypt; 3 Department of Dyeing, Printing and Auxiliaries, Textile Technology Research Institute, National Research Centre, 33 El-Bohouth St. (Former El-Tahrir St.), Dokki, Cairo, 12622 Egypt; 4 Plant Biochemistry Department, Agricultural and Biology Research Institute, National Research Centre, 33 El-Bohouth St. (Former El-Tahrir St.), Dokki, Cairo, 12622 Egypt; 5 Chemistry of Tanning Materials and Leather Technology Department, Chemical Industries Research Institute, National Research Centre, 33 El-Bohouth St. (Former El-Tahrir St.), Dokki, Cairo, 12622 Egypt

**Keywords:** Red pigment, *Talaromyces atroroseus*, Genetic improvement, γ-Rays; UHPLC/HRESI-MS, Skin irritation, Wool fabric dyeing

## Abstract

**Background:**

During the last decade, enormous research efforts have been directed at identifying potent microorganisms as sustainable green cell factories for eco-friendly pigments. *Talaromyces atroroseus* has recently been shown to excrete large amounts of azaphilone mycotoxin-free red pigment mixture comprising some known coloring components together with many uncharacterized metabolites. In this study, a new *Talaromyces atroroseus* isolate was identified via sequencing of the fragment of the nuclear ribosomal gene cluster containing internal transcribed spacers and 5.8S rRNA gene. The parameters that affected the level of pigment production were optimized in uncommon static conditions of culture and genetic improvement, via γ-irradiation, to improve pigment yield. Moreover, chemical characterization using LC/MS and skin safety test of the target pigment mixture were precisely conducted to maximize its benefits as a natural and safe red pigment for wool fabrics.

**Results:**

Molecular identification via the sequencing of the ITS of the rDNA encoding gene cluster revealed that the fungal isolate TRP-NRC was *T. atroroseus* TRP-NRC (deposited in GenBank under accession number MW282329). In the static conditions of culture, pigment production was dramatically enhanced to 27.36 g/L in an optimum yeast malt peptone medium of 2% mannitol at *p*H 2−4.5 and 30 °C for 7 days of incubation. Under exposure to a 400-Gy γ-radiation dose, pigment yield was increased to a 3-fold level higher than that recorded for the wild type. Based on the inter-simple sequence repeats (ISSR), as a molecular marker tool, the wild-type *T. atroroseus* TRP-NRC strain and its mutants were discriminated. The UHPLC/HRESI-MS analytical tool characterized 60 metabolites, including many unknown molecules, at appropriate concentrations. It is worthy to note that four mitorubrin derivatives were identified for the first time in *T. atroroseus*, i.e., mitorubrinolamine acetate, dihydro-PP-O, mitorobrinal, and mitorubrinol. The range of irritation indexes (0−0.1) demonstrated an adequate skin safety after the direct local application of the pigment mixture. Finally, the pigment mixture exhibited a remarkably good dyeing ability in wool fabrics, with high-fastness properties.

**Conclusions:**

Because of its sustainable and economic production, the target red pigment mixture may be applied in the future in textile, food, cosmetics, or different pharmaceutical industries after extensive conventional safety and toxicity studies, which are currently under consideration.

**Supplementary Information:**

The online version contains supplementary material available at 10.1186/s43141-022-00335-2.

## Background

Many synthetic colorants are widely used in different industries, despite their detrimental effects on human health including allergic, carcinogenic, and cytotoxic effects, which pose a threat to all forms of life. Therefore, great attention is currently being paid to natural dyes, because they play some beneficial roles in human health. Several pharmaceutical studies reported significant biological effects for different pigments, e.g. antimicrobial [1, 2], antioxidant [3], and anticarcinogenic properties [4]. Natural pigments are mainly synthesized by plants, animals, and microbes. Since ancient times, indigo, turmeric, annatto, and other plant species have been used as pigments sources. However, because of season dependency, expensive production, and vulnerable plant species loss because of their extensive use, the process of pigment production from plants is not considered a resolved issue [5]. Microorganisms, including fungi, bacteria, and algae, have been shown to be good alternative sources of natural pigments because of the good understanding of their cultural approaches and their pigment production on a large scale. Among the most potent microorganisms that have been reported regarding natural pigment production, *Xanthophyllomyces dendrorhous* and the microalga *Haematococcus lacustris* have the potential for being incorporated in the production of astaxanthin as a carotenoid pigment with health-promoting properties [6–8]. Fungi are considered the best source for pigment production because of their ability to biosynthesize for more soluble and stable pigments via an easy process. The extraction of pigments of several chemical classes, such as melanins, flavins, quinones, phenazines, monascin, carotenoids, azaphilones, and others from fungi has been reported [6]. It is well known that many filamentous fungi, such as *Fusarium*, *Penicillium*, *Trichoderma*, *Monascus*, *Aspergillus*, *Talaromyces*, and *Laetiporus*, are good fungal pigment producers [2, 9]. The *Talaromyces* genus has been reported as a rich microbial source of widespread color-contributing agents [5, 10–12]. Moreover, *T. atroroseus* is a very interesting candidate for the mass production of *Monascus* pigment, because of its capability to produce various mycotoxin-free pigments [13–17]. Several studies have dealt with the chemical characterization of the fungal pigments or their active pure isolates by LC/MS or other tools, e.g., UV, IR, and NMR spectroscopy [10, 12, 18–25]. The limitations in the use of fungi as pigment producers include the co-production of mycotoxins in the growth media; for example, *Fusarium* spp. are β-carotene and lycopene producers that have a restricted drawback in their commercial use in pigment synthesis because of the generation of mycotoxins [8]. The first aim of the current study was to isolate and develop a fungal mycotoxin-free pigment producer of safe applications for human consumption. The different techniques of genetic engineering that have been used for the enhancement of fungal pigments include the modification and alteration of genes [9]. Genetic mutagenesis is considered a powerful tool to manipulate microorganisms; however, it requires prior knowledge and understanding of the biosynthetic mechanisms and targeted genes. Comparably, traditional mutational genetics induced by chemical and physical mutagens does not require a previous understanding of the genetic makeup of specific organisms and is cost effective [26]. A previous study reported by Jia et al. [27] employed ultraviolet-light induced traditional mutagenesis and metabolic engineering through the use of *Agrobacterium tumefaciens*-mediated transformation to eliminate citrinin as a mycotoxin in the pigment-producing fungus *Monascus purpureus* (SM001 strain). Here, the traditional mutational genetics applied using γ-irradiation to improve the production of the pigment in a mycotoxin-free fungal isolate. ^60^Co γ-irradiation, as a highly energetic electromagnetic radiation, has high mutagenic effectiveness because of its high penetration into cells, thus causing genetic mutations [28, 29]. Although this approach is time consuming regarding the selection of target mutants, it has achieved the same aim that can probably be attained via metabolic engineering with cost-effectiveness. Moreover, to recognize genetic variations among species and perform comparative analyses based on genomic DNA, different techniques are currently available that use molecular methods to assess genetic diversity [30, 31], such as RAPD, AFLP, and ISSR. ISSR is an effective molecular marker that has been used to recognize genetic variations among target mutants with a higher pigment yield, comparable to that of the wild-type strain, and that is based on the new arrangement occurring in DNA molecules in response to random mutation using γ-irradiation. This study was the first to use traditional mutational genetics to improve pigment production in a fungal mycotoxin-free pigment producer. The intended use of the natural pigment in the textile industry forced us to analyze the fungal pigment regarding its biodegradable nature, in particular, as the production of safe and sustainable pigments appears to be highly advantageous compared with artificial colorants, which are considered polluting sources to land and water [7]. Moreover, a recent study aimed at the estimation of the dermal toxicity of pigments from several *Thermomyces* strains and *Penicillium**purpurogenum* in Wistar rats demonstrated their non-toxic nature and postulated their intrinsic application in the cosmetics and dyeing industries [32]. The potential coloring application of the fungal pigments has been investigated on different textile substrates such as wool, silk, and cotton [33]. This study aims at the isolation, molecular identification, optimization, and genetic improvement of a new *T. atroroseus* isolate, for potential red pigment production. In addition, new conditions were tried for the LC/HRESI-MS structural characterization of the target pigment, as well as its assessment regarding skin safety and dyeing of wool fabrics (performance and fastness properties).

## Methods

### Isolation and maintenance of a pigment-producing fungus

Three types of samples were collected from Giza governorate, Egypt. The first included soil samples: dry rice and wheat straw were collected randomly from various farms. The second consisted of infected fruit samples: infected wet orange and pomegranate fruits were collected from different marketplaces. The third included fruit wastes: wet orange and carrot drink wastes collected from juice shops. Pigment-producing fungi were isolated via serial dilution (up to 10^−9^) using sterile saline solution. After thorough mixing, a 50-μL aliquot from suitable dilutions was poured, in triplicate, onto YMP agar plates (yeast extract, 3 g/L; malt extract, 3 g/L; peptone, 5 g/L; and glucose, 20 g/L) and incubated for 5 days at 28 ± 0.5 °C. The fungal colonies with red pigmentation were isolated. To obtain an axenic fungal culture, spores were transferred into sterile Falcon tubes containing sterile distilled water, for dilution; subsequently, 50 μL of the appropriate dilution was placed on YMP agar plates in three replicates. The pure culture was maintained on YMP agar slants at 4 °C for further studies, with the laboratory code of TRP-NRC.

### Morphological identification

The promising fungal isolate was assessed for colony morphology. The colony characteristics and microscopic features, such as conidia, conidiophores, and size and shape of hyphae, were examined using a light microscope (Olympus CH40RF200, Japan) through slide cultures [34]. The morphological features of the fungal isolate were also observed using scanning electron microscopy (FEI, model: Quanta FEG 250, Netherlands). The preparation of the samples was carried out according to Salvia et al. [35].

### Molecular identification of the red-pigmented fungal isolate and phylogenetic tree construction

Genomic DNA was isolated from the TRP-NRC isolate using the DNeasy Plant Mini Kit (Qiagen, Maryland, USA), according to the manufacturer^’^s instructions. The universal primers ITS1 (5′-CCGTAGGTGAACCTGCGG-3′) and ITS4 (5′-TCCTCCGCTTATTGATATGC-3′) were used to amplify and sequence the nuclear ribosomal ITS 1 and 2, as well as the gene encoding the 5.8S rRNA (ITS1-5.8SrRNA-ITS2 fragment) [36]. The PCR conditions consisted of an initial denaturation step for 5 min at 94 °C; followed by 35 cycles of denaturation for 30 s at 94 °C, annealing for 30 s at 55 °C, and amplification for 1 min at 72 °C; with a final extension step at 72 °C for 10 min. The amplicon obtained by PCR was purified using the QIA quick PCR purification kit (Qiagen, Maryland, USA). The purified PCR product was subjected to sequencing via the Sanger sequencing method using a 3500 genetic analyzer, and a big dye X terminator kit (Thermo Fisher, USA) in the forward and reverse directions in a biomedical laboratory of colors (Clinilab, Egypt). The sequences were edited using the BioEdit 7.1.10 software [37], and BLASTn was used to detect similarities with other relatives [38]. Multiple sequence alignments of the ITS sequence from this study and ITS sequences retrieved from GenBank were performed using the MUSCLE [39] algorithm available in MEGA X [40]. The evolutionary history was inferred using the neighbor-joining method [41] with a 1000 bootstraps run [42], and the evolutionary distances were computed using the Jukes−Cantor method [43]. This analysis involved 19 nucleotide sequences.

### Optimization of the culture conditions for pigment production

Different factors were examined to determine the optimum culture conditions for maximum red pigment production from TRP-NRC isolate, such as carbon source, *p*H range, various medium volumes, and inoculation type.

#### Inoculum seed preparation

Spores of 5-day-old fungal culture slants were obtained by addition of 10 mL sterilized water, where the fungal growth was crushed with culture loop in presence of 0.01% Tween 80 and vortex. The spore suspension (1%) was used to inoculate 250-mL conical flasks containing100 mL of the pigment water extract constituted from the YMP culture medium. Afterward, incubation was done on a rotatory shaker at 150 rpm and static conditions for 7 days at 30^o^C.

#### Carbon sources

Different carbon sources were examined such as glucose, fructose, sucrose, galactose, mannitol, xylose, and glycerol. Briefly, 2% (w/v) of different carbon sources was added to the YMP medium and incubation was run under the same conditions.

#### Effect of pH

The pigment-production medium was adjusted to various *p*H values, ranging from 2.0 to 7.5 using diluted HCl and NaOH solutions. The flasks were incubated under static conditions at 30 °C for 7 days.

#### Effect of inoculum type

The flasks of the pigment medium were divided into two sets. The first set was inoculated with fungus spores and the second with fungus mycelium, followed by incubation of all flasks in the conditions described above.

#### Effect of medium volume

Various volumes of pigment medium (25–200 mL) were introduced in 250-mL conical flasks and sterilized by autoclaving with fungus mycelium. All experiments were incubated under the conditions described above, in three replicates.

### Pigment extraction

At the end of the fermentation period, the experimental flasks were placed on a rotatory shaker at 150 rpm for 30 min. The whole culture was simply filtered using Whatman No. 1 filter paper, followed by centrifugation for 15 min at 10,000 rpm. The supernatant, as the source of extracellular pigments, was considered for evaluation. The investigated red pigment mixture had maximum absorbance at 500 nm during a full scanning of its solution at 200−900 nm. Thus, the pigment content was estimated *by measuring the absorbance of the filtered *pigment extract at λ_max_ of 500 nm on a UV-visible spectrophotometer* (*V630, JAVSCO, Japan) with 10 mm path length quartz cuvettes at room temperature*, *while checking for variations in all conditions. The extracts of the red pigment were stored in the dark at 4 °C before use in subsequent tests.

### Mutation induction by γ-rays and screening of pigment hyper-producing mutants

A 3-day-old grown slant of the highest pigmented fungal strain was exposed to different doses of γ-irradiation (200, 400, 600, and 800 Gy). The process was carried out at the Gamma chamber 4000 A Co-60 irradiation facility (Bhabha Atomic Research Centre, India), at a dose rate of 1.915 kGy/h (Egyptian Atomic Energy Authority, Nasser City, Egypt). Because the main goal of γ-irradiation is obtaining red pigment hyper-producing mutant strains, its effect on the survival of fungal stain was neglected. Serial dilution was done on the YMP medium for screening hyper-producing mutants via cultivation at optimized conditions. The non-irradiated strain was used as a test control.

### Molecular differentiation of mutants using ISSR amplification

Twelve primers (Metabion Corporation, Germany) were used to amplify DNA with polymorphic and monomorphic bands (Table 1). ISSR amplifications were performed in 25-μL reactions consisting of 2 μL of 10 μmol primers, 2 μL of DNA template, 12.5 μL of PCR Master Mix 2× concentration (Thermo Scientific, USA), and 8.5 μL of dd H_2_O. The PCR conditions were according to the literature [44], with some modifications, as follows: initial denaturation at 95°C for 3 min; followed by 35 cycles of 30 s at 94 °C, 30 s at 45 °C, 2 min at 72 °C; and a final extension of 15 min at 72 °C, using a GeneAmp PCR System 2400 Thermal cycler (Perkin-Elmer Norwalk, Connecticut, USA). The amplification products were resolved by electrophoresis on a 1.5% agarose gel containing ethidium bromide (0.5 μg/mL) in 1X TBE buffer at 95 V. PCR products were visualized under UV light and photographed using a Gel Documentation System (BIO-RAD XR^+^ Gel Doc life science research, Bio-Rad Laboratories, Dubai, UAE).

**Table 1:** ISSR primers name and sequences

Primer	Sequence	Primer	Sequence
ISSR-1	5′-AGAGAGAGAGAGAGAGYC-3′	ISSR-7	5′-AGAGAGAGAGAGAGAGYT-3′
ISSR-2	5′-AGAGAGAGAGAGAGAGYG-3′	ISSR-8	5′-CTCCTCCTCCTCCTCTT-3′
ISSR-3	5′-ACACACACACACACACYT-3′	ISSR-9	5′-CTCTCTCTCTCTCTCTRG-3′
ISSR-4	5′-ACACACACACACACACYG-3′	ISSR-10	5′-TCTCTCTCTCTCTCTCA-3′
ISSR-5	5′-ACACACACACACACACYA-3′	ISSR-11	5′-HVHCACACACACACACAT-3′
ISSR-6	5′-ACACACACACACACACYC-3′	ISSR-12	5′-HVHTCCTCCTCCTCCTCC-3′

### PCR-ISSR data analysis

The banding patterns generated by the ISSR marker analyses were compared to determine the genetic relatedness of the samples under study. Clear and distinct amplification products were scored as “1” for presence and “0” for the absence of bands. Bands with the same mobility were scored as being identical. The genetic similarity coefficient (GS) between two genotypes was estimated according to the Dice coefficient [45]*.*

### UHPLC/HRESI-MS method, instrument, and conditions

The LC/MS measurements were carried out through a service type of metabolomics analysis using ESI-MS positive and negative ion acquisition modes on a XEVO TQD triple quadruple mass spectrometer (Waters Corporation, Milford, MA01757 USA). Mass function: MS1; source voltages: capillary voltage of 3.0 kV and cone of 30 V; Source temperatures (^o^C): desolvation temp. of 440; Source gas flow (L/h): desolvation 900 and cone 50. This system included an UHPLC interface with a column: ACQUITY UPLC-BEH C18 (1.7 μm–2.1 × 50 mm), a flow rate of 0.2 mL/min, injected volume: 10 μL of 1 μg/μL solution, and a gradient solvent system consisting of (A) 0.1% formic acid in H_2_O and (B) 0.1 % formic acid in CH_3_CN solutions (Table 2).

**Table 2:** Time program of UPLC/HRESI-MS analysis of *T. atroroseus* TRP-NRC red pigment

Time/min	0	2	5	15	22	25	26	29	32
%A	90.0	90.0	70.0	30.0	10.0	10.0	0.0	0.0	90.0
%B	10.0	10.0	30.0	70.0	90.0	90.0	100.0	100.0	10.0

Mobile phase composition, A: 0.1% formic acid in H_2_O and B: 0.1 % formic acid in CH_3_CN

### Skin irritation experiment

This experiment was carried out in accordance with a method published previously, with minor variations, using Wistar male rats (140–150 g, about 3 months in age). Accordingly, two sets of rats*,* each including six animals, were employed. The dorsal side of each rat (1 cm from the midline of the vertebral column) was shaved and labeled before the test [46]. The 1st set was left untreated, to be used for comparison. The target pigment (100%) was applied to the processing set. Immediately after, the area was covered with dressing gauze, over which a plastic sheet was placed. The covering was loosely held in contact with the skin by means of a non-irritating adhesive tape and tied across the diameter of the back of the rats with an elastic bandage. After an exposure period of 24 h, the elastic bandage, the adhesive plaster, the plastic sheet, and the gauze were carefully removed to avoid skin damage, followed by rinsing of the test site with distilled water. The animals were then examined for the presence of erythema and edema according to the Draize dermal irritation scoring system at 1-, 24-, 48-, and 72-h intervals [47].

### Dyeing processing of wool fabric

#### Dyeing procedures

A set of lab-scale dyeing experiments were carried out on wool fabrics (Twill weave fabric of 310 g/m, Golden Tex Co., Tenth of Ramadan City, Egypt) in an IR dyeing machine using the produced pigment without mordanting [48]. The dye bath was prepared with the target pigment (20–100%) at a liquor ratio of 20:1 with 1 g/L of leveling agent (Lyogen NH liquid; Archroma) and a *p*H range of 3−7. Dyeing was started at 40 °C for 10 min, then the dyeing bath temperature was raised gradually to 90 °C over 30 min and the process continued for 45 min. After dyeing, the temperature was lowered to 60 °C, then the dyed samples were rinsed with distilled water and dried at room temperature.

#### Pigment exhaustion, color measurements, and fastness properties

The pigment uptake by the wool fabrics was measured by sampling the dyebath before and after dyeing. The pigment absorbance (A) of the dyebath was measured on a UV2401PC UV/VIS spectrophotometer (Shimatzu, Japan) at *λ*_max_ of 499 nm. The percentage of dyebath exhaustion (%E) was calculated using equation-1.

1 $$\%E=\left[1-\left(A2/A1\right)\right]\times 100$$

where *A*_1_ and *A*_2_ are the absorbances of the pigment before and after dyeing, respectively.

After dyeing, the samples were washed off for 15 min at 60 °C using an aqueous solution of 2 g/L of non-ionic detergent (Sera Wash M-SF, DyStar, Egypt), then rinsed with distilled water. The color strength (*K*/*S*) and colorimetric data of the pigmented fabrics were measured using an UltraScan PRO spectrophotometer (Hunter Lab, USA) under illuminant D65, 10° standard observer. The *K*/*S* values of pigmented fabrics were measured by the light repentance technique using the Kubelka−Munk equation (Eq. 2) [49].

2 $$K/S=\left(1-R\right)2/(2R)$$

where *R* is the decimal fraction of the reflection of the dyed fabric, *K* is the absorption coefficient, and *S* is the scattering coefficient*.*

Color readings were expressed in the CIELAB color space system (often denoted as L*, a*, b*, C* and *h*^*°*^ coordinates). Thereafter, the color fastness was tested according to the ISO standards in samples treated with pigment concentrations of 20 and 80% [50]. Examination of the surface morphology of the dyed wool fabric was performed using a scanning electron microscope (SEM), where samples were mounted onto carbon adhesive tabs on aluminum stubs, coated with gold using Quorum Q 150 ES (UK) for 60 sec with a layer 20 nm and analyzed at 20 kV with SEM magnification of 2.00 kx on a TESCAN VEGA 3 scanning electron microscope (Czech Republic).

## Results

### Isolation and molecular identification

**Fig. 1:**
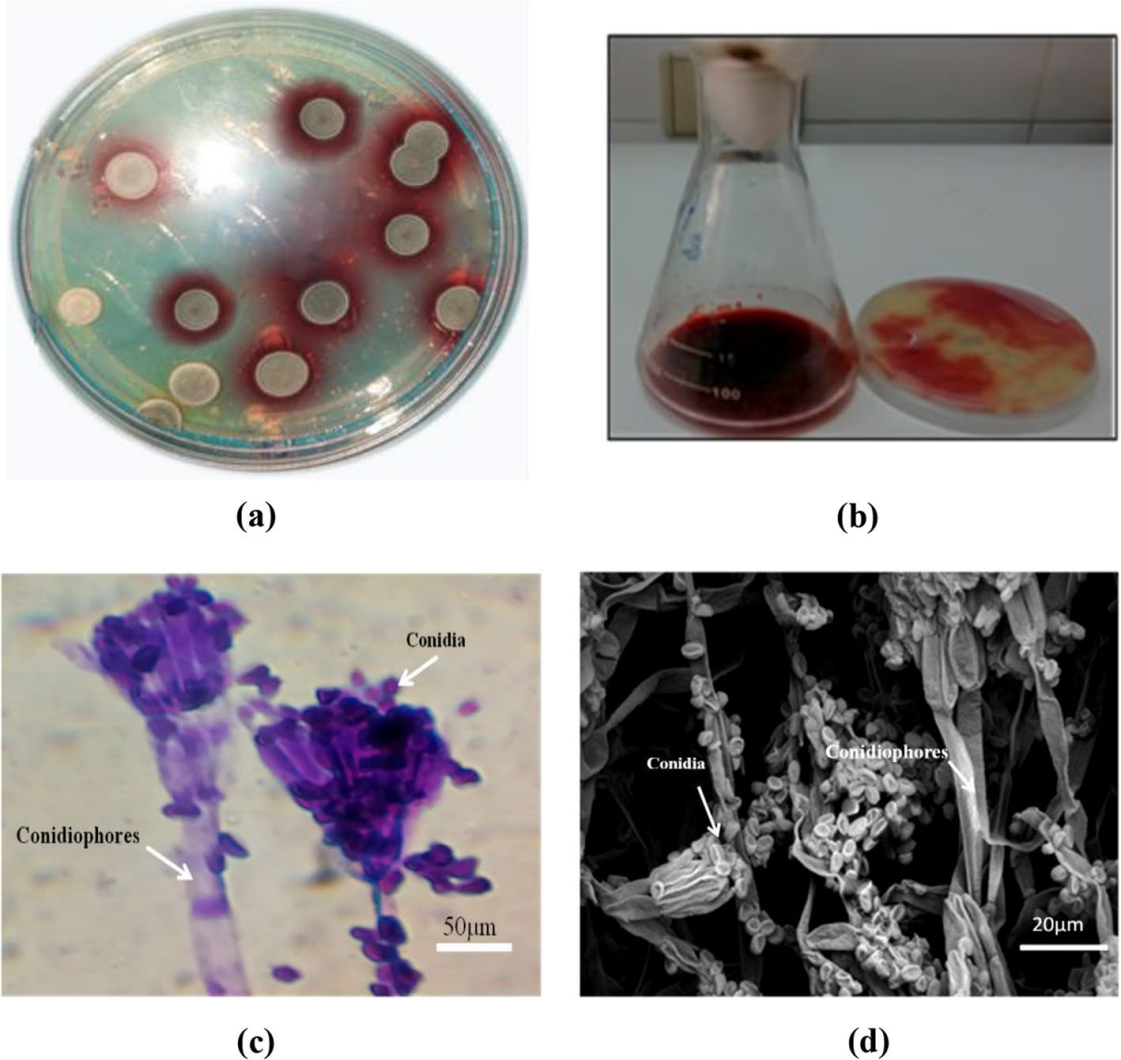
Morphological characterization of *T. atroroseus* TRP-NRC isolate: **a** colony morphology after five days on YMP plates, **b** diffusible red pigment production in the media, **c** morphology of conidiophores carrying spores under light microscope, **d** morphology of conidiophores carrying spores under electro scanning microscope

Figure 1 shows the morphology of the pigment-producing fungal isolate on YMP media after culturing for 5 days at 28 ± 0.5°C. Only one morphological characteristic of red pigment-producing fungi was observed, and the isolate was labeled with the code TRP-NRC. Morphologically, the fungal colony was characterized by a diameter of 12–15 mm after 5 days of growth on YMP plates, and became a grayish–green color, depending on the sporulation state, with white borders (Fig. 1a). The culture produced a diffusible red pigment that was observed in the media (Fig. 1b). Thick-walled ellipsoidal conidia with a rough to finely roughened appearance were reported; conidiophores are typically branching, biverticillate, or sometimes have sub-terminal branches (Fig. 1c, d). Based on the abovementioned characteristics, the TRP isolate was expected to belong to the *Talaromyces* genus. The morphology-based identification was further supported by molecular-based identification, which provides further taxonomic details at the species level.

### Molecular identification of the red-pigmented fungal isolate

**Fig. 2:**
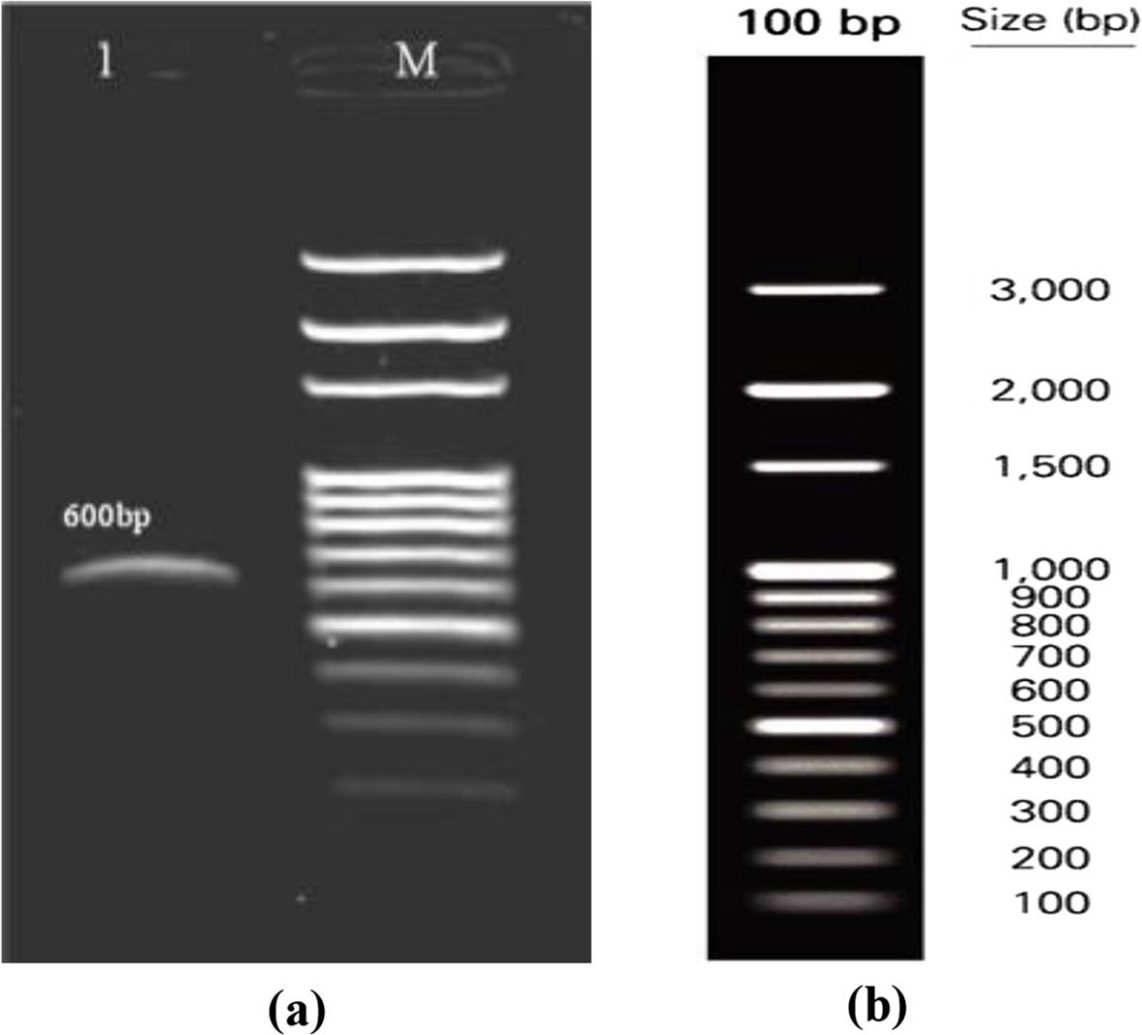
**a** Agarose gel electrophoresis for PCR product of internal transcribed spacer (ITS) of *T. atroroseus* TRP-NRC isolate; (1): molecular length of PCR amplified band of ITS1-5.8SrRNA-ITS2 Fragment; M: DNA marker, 100 base pair (bp) enzynomics, **b** Enzynomics_DM001_100 bp DNA Ladder Marker

The ribosomal internal transcribed spacer (ITS1-8.5S-ITS2) rDNA of the fungal isolate was successfully amplified using conventional PCR and observed as a clear band of ~ 600 bp (Fig. 2). The obtained sequence had 100% similarity with *T. atroroseus* strain DTO 390-I4. The fungal isolate identified in this study was submitted to the NCBI GenBank database as *T. atroroseus*, strain TRP-NRC under accession number MW282329.

**Fig. 3:**
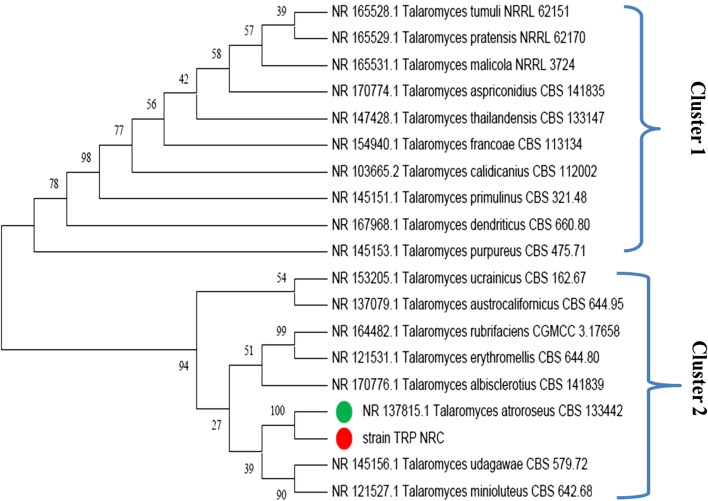
Phylogenetic evolutionary relationship of *Talaromyces atroroseus* TRP NRC and other related sequences retrieved from Genbank based on ITS1- 5.8S-ITS2 region using MEGA X. numbers above nodes indicate percentages of their bootstrap support

In this study, based on the sequence of the ITS region, a *T. atroroseus* strain TRP-NRC phylogenetic analysis was performed. The comparison between this isolate and the ITS recorded sequences that were retrieved from NCBI ensured the identification of the isolate at the genus and species level. The constructed phylogenetic tree illustrated two major clusters. The first cluster included 10 ITS regions and belongs to the genus of *Talaromyces*, which showed a close relationship with each other, with different bootstrap values. The second cluster included the fungal isolate, *T. atroroseus* TRP-NRC, which showed a close relationship with *Talaromyces atroroseus* CBS 133442 (Acc no: NR 137815.1), in the same clade, with a bootstrap value of 100%, as presented with others in the cluster (Fig. 3).

### Optimization of the culture conditions for best pigment production

It is worth mentioning that no pigment was observed throughout the incubation period after the cultivation of fungal spores and mycelium on the pigment-production medium by shaking at 150 rpm at 30°C for 7 days, whereas a red pigment was noticed on solid-state fermentation (SSF).

#### Effect of the carbon source

**Fig. 4:**
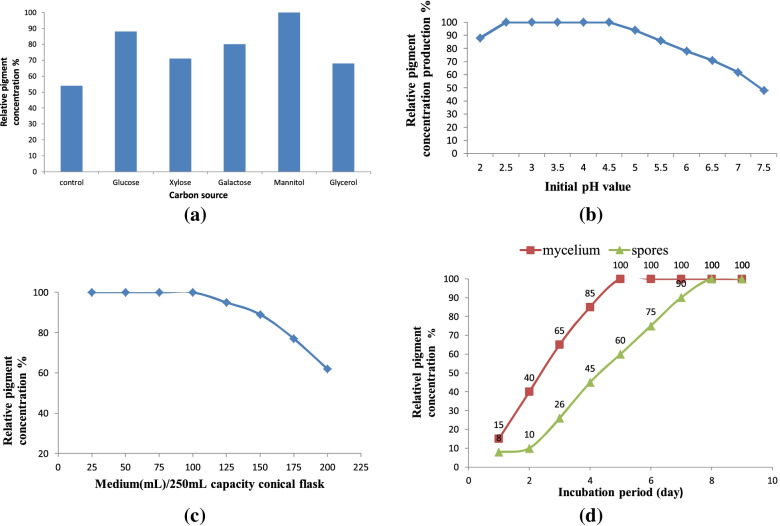
Parameters affecting the optimization for the pigment production of *T. atroroseus* TRP–NRC, under static condition at 30^o^C after 7 days incubation: **a** Effect of carbon source, **b** Effect of initial pH value, **c** Effect of medium volume/fermentor capacity, **d** Effect of inoculums type

The use of mannitol as a carbon source yielded the highest pigment concentration, which was scored as 100%, followed by glucose (88%) (Fig. 4a). Simultaneously, in the presence of glycerol, the lowest concentration of red pigment was achieved. The concentration of the pigment in the liquid medium released by the fungus was measured at a *λ*_max_ of 500nm. This study achieved an economic medium that supported enhanced pigment production, i.e., YMP supplemented with 2% mannitol as a carbon source.

#### Effect of pH value

The maximum production of pigment was achieved at a *p*H range of 2–4.5, whereas it was decreased below or above this range (Fig. 4b).

#### Effect of medium volume

Under static fermentation conditions, the highest pigment production was obtained while adopting 50% capacity of the fermentation medium in fermentation vessels (Fig. 4c).

#### Effect of inoculum type

The use of the fungal mycelium for inoculation of the pigment-production medium exhibited an advantage regarding the acceleration of pigment production by decreasing the fermentation time, to produce the maximum pigment yield (Fig. 4d).

### Mutation induction using γ-radiation

**Fig. 5:**
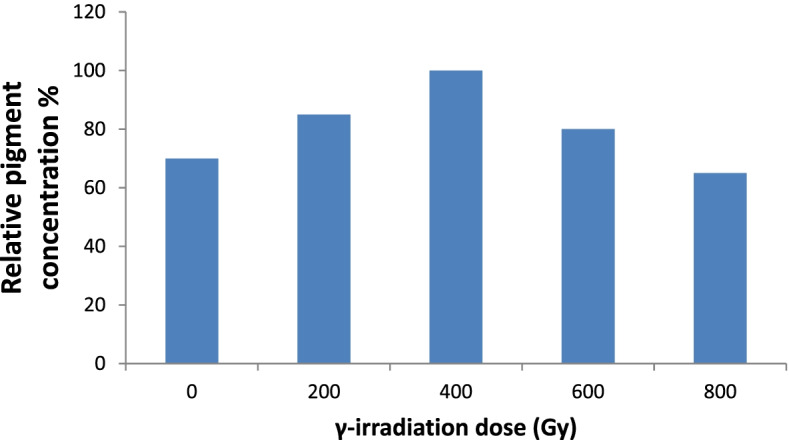
Effect of the γ-irradiation on the yield concentration of the total red pigment in the *T. atroroseus* TRP-NRC water extract

A dose of 400 Gy led to a 30% increase in pigment concentration, which was the maximum pigment production under optimized conditions compared with the unexposed wild-type fungus (Fig. 5). Conversely, the lowest concentration was observed at a dose of 800 Gy.

### Molecular differentiation, genetic distance, and cluster analysis between *T. atroroseus* and its mutants using the PCR-ISSR technique

**Fig. 6:**
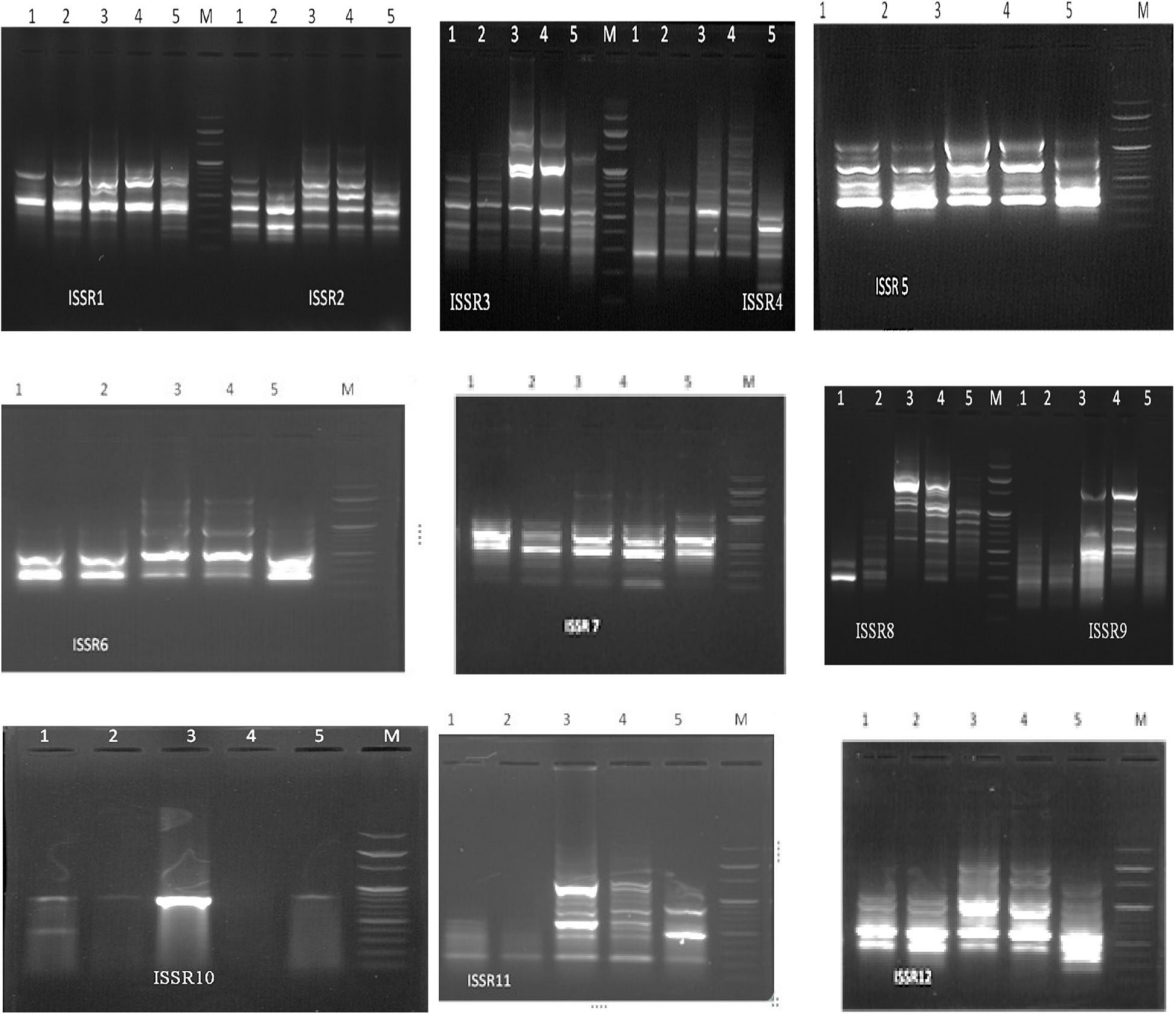
Agarose gel electrophoresis for PCR products of ISSR primers for *T. atroroseus* TRP-NRC (1) and its mutants (2–5). (M): M: 100 bp DNA marker (enzynomics)

PCR-ISSR gave a total of 143 clear and reproducible fragments ranging between 4 (in ISSR-10) and 17 (in ISSR-3), with an average size of 132–1880 bp across the wild-type strain and its mutants (Fig. 6). The ISSR-3 primer was considered being the most efficient, as it succeeded in amplifying the highest numbers of bands; moreover, it exhibited a higher discriminatory value because of the high number of polymorphic bands generated by this primer relative to the total bands generated by all primers.

**Fig. 7:**
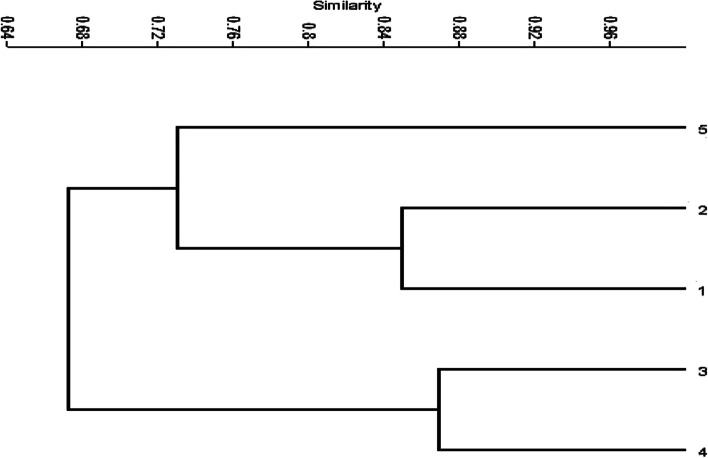
The genetic dendrogram of genetic fingerprint and relationships between *T. atroroseus* TRP-NRC **(**1) and its mutants (2–5) revealed by their ISSR markers

Based on the ISSR banding profile, a cluster dendrogram was obtained (Fig. 7). In terms of the similarity coefficient, the similarity between the wild-type *T. atroroseus* (no. 1) and its four mutants (M_200_ (no. 2), M_400_ (no. 3), M_600_ (no. 4), and M_800_ (no. 5)) was in the range of 63–85%. The dendrogram that was constructed using UPGMA illustrated two distinct groups: the first one exhibited a similarity of 85 and 73% for M_200_ (no. 2) and M_800_ (no. 5), respectively, with *T. atroroseus* as the wild-type strain (no. 1). Conversely, the second group including M_400_ (no. 3) and M_600_ (no. 4) exhibited a similarity of 63 and 67%, respectively, relative to the wild-type strain.

### UHPLC/HRESI-MS analysis of the total red pigment from *T. atroroseus* TRP NRC

**Fig. 8:**
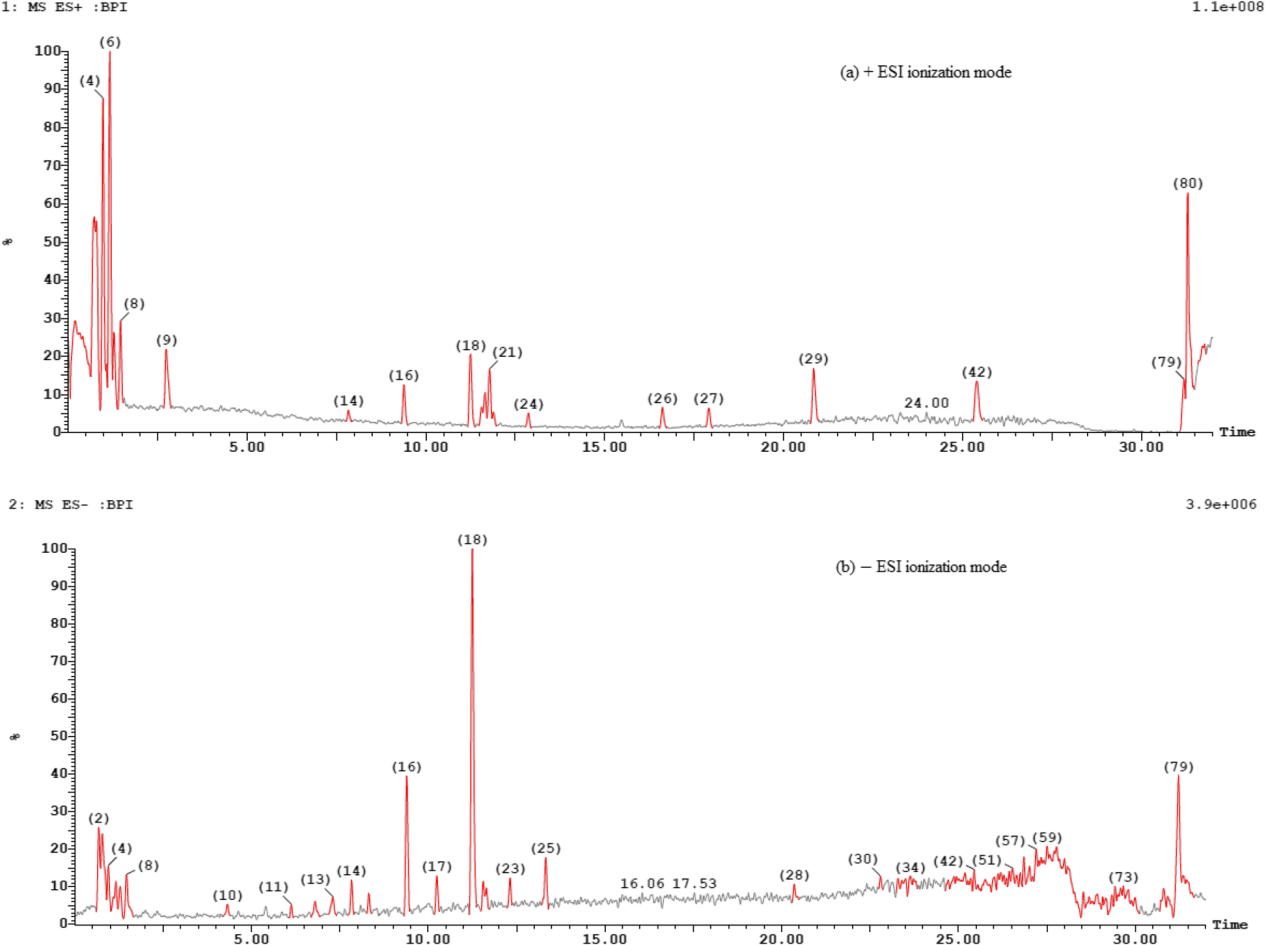
TIC-chromatograms of LC/HRESI-MS analysis for the total pigment of *T. atroroseus* TRP-NRC water extract: **a** positive ESI; **b** negative ESI modes of ionization

**Fig. 9:**
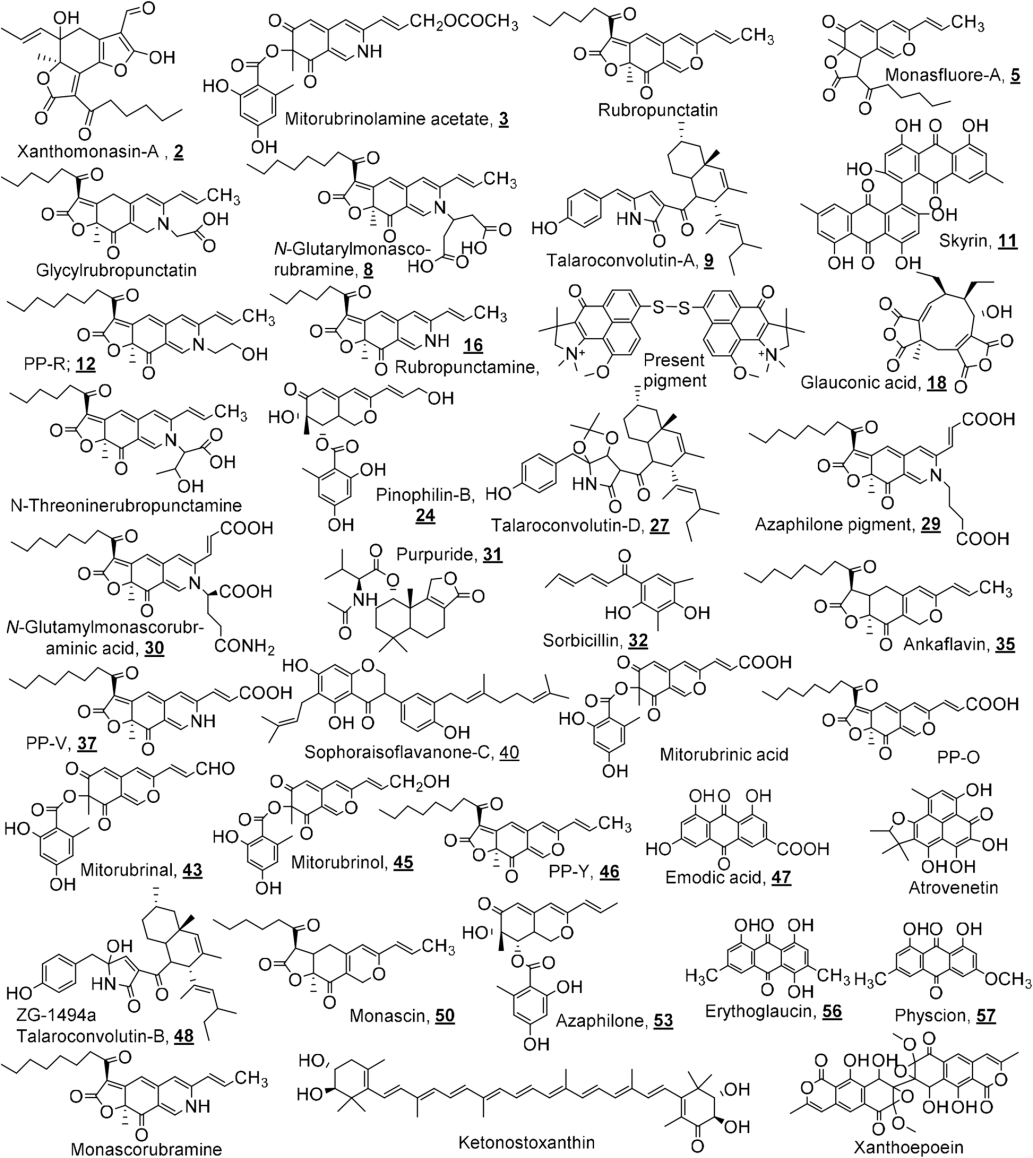
Structural formulas for some metabolites identified in the total pigment of *T. atroroseus* TRP-NRC water extract

Using both modes of ionization, UHPLC/HRESI-MS led to the tentative identification of 60 known metabolites (MWs range 250−650 amu) along with the recording of the *pseudo*-molecular ion peaks of many unknown ones (MW ranges: ˂ 200 and 700−950 amu). From the output data of the TIC (Fig. 8a, b) and corresponding MS-spectra (Fig. S1), a total of 24 and 31 metabolites are identified in the positive and negative modes, respectively (Table 3, Fig. 9). Some metabolites are detected in reasonable abundances in both modes (3,9,11,12,13,17,28,29); simultaneously, adduct ions like [M+Na]^+^, [M+K]^+^ or [M+HCO_2_]^−^ have showed confirmative masses for other metabolites (3,31,36,38,41,48,59). Moreover, many metabolites are co-eluted in the majority of peaks although the high efficiency of the separation under the conditions used due to the crowding of the total pigment extract (Table 3 & Figs. 8, S1). Among the peaks recorded, TIC showed 6 major peaks in positive mode (ID: 1, 3−6 & 80, Fig. 8a), while three are recorded in the negative one (ID: 16, 18 & 79, Fig. 8b). Four metabolites are identified as mitorubrinolamine acetate (3), dihydrorubropunctatin (4), monasfluore-A (5) and talaroconvolutin-A (9) with unknown 907.8774, 923.8765 [M+H]^+^ in the positive TIC, while as negative ions derivatives of *N*-threoninerubro-punctamine (22) and talaroconvolutin-A (28), azaphilone pigment (29) with unknown 929.9794, 930.0319, 945.9055 [M−H]^–^ were recorded. The common metabolites identified before in *Talaromyces* spp., especially in *T. atroroseus* are detected on 18 peaks of medium abundances, 10 (ID: 1, 7−9, 16, 18, 21, 29, 42 & 79) and 8 (ID: 2−4, 8, 14, 17, 23 & 25) peaks were recorded in the positive and negative TICs, respectively.

**Table 3:** Chemical structures and exact masses of the metabolites identified in the pigment water extract of *T. atroroseus *TRP NRC by UHPLC/HRESI-MS

Peak ID	***R***_***t***_, min	Found monoisotopic mass	MW, amu = Da	MF	Chemical name
**2**	0.68	272.9545 [M−H]^−^	274.0590	C13H10N2O5	(*E*)-3-oxo-3-((1-(4-oxo-4H-Benzo[e][1,3]oxazin-2-yl)ethylidene)amino)propanoic acid, **1**
**2**		387.0887 [M−H]^−^	388.1522	C21H24O7	Xanthomonasin-A, **2**
**3**	0.74	438.1913 [M−H]^−^	439.1267	C23H21NO8	Mitorubrinolamine acetate, **3**
		440.2273 [M+H]^+^			
**4**	0.98	440.2050 [M+H]^+^			
		462.1462 [M+Na]^+^			
		438.2209 [M−H]^−^			
**4**		357.1845 [M+H]^+^	356.1624	C21H24O5	Dihydrorubropunctatin, **4** or Monasfluore-A, **5**
**4**		190.9803 [M−H]^−^	192.0899	C10H12N2O2	Dihydrochrysogine, **6**
**4**		412.1799 [M−H]^−^	413.1838	C23H27NO6	Dehydroglycylrubropunctatin, **7**
**4**		510.2267 [M−H]^−^	511.2206	C28H33NO8	*N*-Glutarylmonascorubramine, **8**
**6**	1.17	488.1899 [M+H]^+^	487.3086	C32H41NO3	Talaroconvolutin-A, **9**
		486.2599 [M−H]^−^			
**6**		119.9371 [M+H]^+^	119.0219	C3H5NO4,	β-Nitropropanoic acid, **10**
**7**	1.28	488.2377 [M+H]^+^	487.3086	C32H41NO3	Talaroconvolutin-A, **9**
		486.2180 [M−H]^−^			
**7**		539.3469 [M+H]^+^	538.0900	C30H18O10	Skyrin, **11**
**8**	1.47	424.3120 [M−H]^−^	425.2202	C25H31NO5	PP-R; 7-(2-Hydroxyethyl)monascorubramine, **12**
		426.2246 [M+H]^+^			
**8**		428.3271 [M−H]^−^	429.2515	C25H35NO5	Tetrahydro-PP-R, **13**
		430.1977 [M+H]^+^			
**9**	2.75	227.0675 [M+H]^+^	226.1205	C12H18O4	2,4-Dihydroxypentyl)-2-methylbenzene-1,3diol, **14**
**11**	6.13	470.4424 [M−H]^−^	471.2773	C31H37NO3	Deoxytalaroconvolutin-A, **15**
**12**	6.80	352.1925 [M−H]^−^	353.1627	C21H23NO4	Rubropunctamine, **16**
**14**	7.84	697.4280 [M+H]^+^	696.2692	C40H44N2O5S2	Hydrooxy-present pigment, **17**
		695.4290 [M−H]^−^			
**14**		349.3371 [M+H]^+^	348.1209	C18H20O7	Glauconic acid, **18**
**15**	8.32	502.1199 [M−H]^−^	503.3036	C32H41NO4	Dehydrotalaroconvolutin-B
					Dehydro ZG-1494a, **19**
**16**	9.38	390.1997 [M+H]^+^	389.2202	C22H31NO5	Dehydro-Purpuride, **20**
**16**		430.2012 [M+H]^+^	429.2515	C25H35NO5	Tetrahydro-PP-R, **21**
**16**		452.2859 [M−H]^−^	453.1788	C25H27NO7	Dehydro-*N*-threoninerubropunctamine, **22**
**17**	10.24	221.2123 [M−H]^−^	222.0892	C12H14O4	1-(3,5-Dihydroxy-4-methylphenyl)pentane-2,4-dione, **23**
**17**		401.1828 [M−H]−	402.1315	C21H22O8	Pinophilin-B, **24**
**17**		472.3087 [M−H]^−^	473.2930	C31H39NO3	Demethyltalaroconvolutin-A, **25**
**17**		494.3125 [M−H]^−^	495.1893	C27H29NO8	Dehydroazaphilone pigment, **26**
**17**		562.2547 [M−H]^−^	563.3611	C35H49NO5	Talaroconvolutin-D, **27**
**18**	11.26	476.2532 [M+H]^+^	475.3086	C31H41NO3	Demethyl-dihydrotalaroconvolutin-A, **28**
**18**		474.2838 [M−H]^−^			
**18**		498.2573 [M+H]^+^	497.2050	C27H31NO8	Azaphilone pigment, **29**
		496.3168 [M−H]^−^			
**19**	11.55	539.2065 [M−H]^−^	540.2108	C28H32N2O9	N-glutamylmonascorubraminic acid, **30**
**19**		392.1892 [M+H]^+^	391.2359	C22H33NO5	Purpuride, **31**
		414.2019 [M+Na]^+^			
**20**	11.66	436.2874^− ^[M+HCO_2_]^−^			
**20**		233.1178 [M+H]^+^	232.0199	C14H16O3	Sorbicillin, **32**
**19**		293.1682 [M−H]^−^	294.1256	C19H18O3	3-Hydroxy-1,8,8,9-tetramethyl-8,9-dihydro-4H-phenaleno[1,2-b]furan-4-one (Atrovenetin derivative, **33**)
**20**		293.1252 [M−H]^−^	294.0852	C13H14N2O6	2-(2-(2-Carboxyacetamido)propanamido)-benzoic acid,(Atrovenetin metabolite, **34**)
**20**		385.1713 [M−H]^−^	386.2093	C23H30O5	Ankaflavin, **35**
**21**	11.78	274.2042 [M+H]^+^	273.0750	C13H11N3O4	The oxidized form of (E)-3-oxo-3-((1-(4-oxo-1,4-dihydroquinazolin-2-yl)ethylidene)-amino)-propanoic acid (Atrovenetin metabolite, **36**)
		296.2686 [M+Na]^+^			
**22**	11.90	412.2079 [M+H]^+^	411.1682	C23H25NO6	PP-V pigment, **37** or purpactin C
**22**		318.3012 [M+H]^+^	317.0648	C14H11N3O6	(*E*)-2-((1-(4-oxo-1,4-Dihydroquinazolin-2-yl)ethylidene)carbamoyl)malonic acid, **38**
		340.3239 [M+Na]^+^			
**22**		230.1811 [M+H]^+^	229.0851	C12H11N3O2	(E)-*N*-(1-(4-oxo-1,4-Dihydroquinazolin-2-yl)ethylidene)acetamide, **39**
**23**	12.33	475.4114 [M−H]^−^	476.2563	C30H36O5	Sophoraisoflavanone-C, **40**
**24**	12.87	415.1910 [M+H]^+^	414.1679	C23H26O7	Dihydro PP-O
		437.1733 [M+Na]^+^			Or Purpactin-A & B, **41**
		453.1935 [M+K]^+^			
**24**		617.3195 [M+H]^+^	616.4128	C40H54O5	Dihydro-4-ketonostoxanthin, **42**
**25**	13.33	395.2523 [M−H]^−^	396.0845	C21H16O8	Mitorubrinal, **43**
**25**		327.1714 [M−H]^−^	328.0947	C18H16O6	(*R*)-3,7-Dihydroxy-1,8,8,9-tetramethyl-8,9-dihydro-4H,6H-benzo[*de*]furo[2,3g]isochromene-4,6-dione, **44**
**26**	16.62	399.2009 [M+H]^+^	398.1002	C21H18O8	Mitorubrinol, **45**
**26**		383.1822 [M+H]^+^	382.1780	C23H26O5	PP-Y; Monascorubrin, **46**
**26**		301.0924 [M+H]^+^	300.0270	C15H8O7	Emodic acid, **47**
**27**	17.91	506.3542 [M+H]^+^	505.3192	C32H43NO4	Talaroconvolutin-B, **48**
		528.3842 [M+Na]^+^			(ZG-1494a)
					Two stereoisomers at C-5 or 26
**28**	20.36	339.2800 [M−H]^−^	340.1311	C20H20O5	Dehydroatrovenetin, **49**
**29**	20.84	359.2497 [M+H]^+^	358.1780	C21H26O5	Monascin, **50**
**29**		239.1775 [M+H]^+^	238.1722	C18H22	2,3,9,10-Tetramethyl-1,2,3,4-tetrahydroanthracene, **51**
**30**	22.79	589.1694 [M−H]^−^	590.1061	C30H22O13	Deoxyxanthoepoein, **52**
**30**		385.0434 [M−H]^−^	386.1366	C21H22O7	Azaphilone, **53**
**32**	23.40	613.1418 [M−H]^−^	614.1636	C30H30O14	Octahydroxanthoepoein, **54**
**34**	23.64	403.3223 [M−H]^−^	404.2199	C23H32O6	Hydrolytic product of Ankaflavin, **55**
**39**	25.19	283.2244 [M−H]^−^	284.0685	C16H12O5	Erythroglaucin, **56**
**40**	25.28	283.1718 [M−H]^−^	284.0685	C16H12O5	Physcion, **57**
**41**	25.35	384.1975 [M−H]^−^	385.2253	C23H31NO4	Tetrahydromonascorubramine, **58**
**42**	25.41	391.2597 [M+H]^+^	390.1234	C21H23ClO5	Sclerotiorin, **59** (Orange azaphilone pigment)
		413.2316 [M+Na]^+^			
**52**	26.65	357.0226 [M−H]^−^	358.1053	C19H18O7	Norherqueinone, **60**

*Rt* retention time, *MW* molecular weight, *MF* molecular formula, *amu/Da* atomic mass unit/Dalton. Underlined bold numbers (**1**–**60**) refer to a serial numeration of the metabolites identified

### Skin irritation test

It is performed to confirm the safety of the target pigment for human skin on textile dyeing. The degree of erythema (reddening of the skin) and edema (inflammation and contact dermatitis) scores were determined. The observed results clarify that all the animals were devoid of any irritation potential and no edema formation was observed in any rat after all time intervals of 1, 24, 48, and 72 h (Table 4). The findings revealed that a value of skin irritancy score lies in the range of 0 to 0.1.

**Table 4:** Effect for the application of *T. atroroseus* TRP-NRC pigment on Skin irritation score

Skin irritation scores of pigment (A = erythema formation score; B = oedema formation score)								
Time, h	1		24		48		72	
**Rats**	**A**	**B**	**A**	**B**	**A**	**B**	**A**	**B**
**1**	0	0	0	0	0	0	0	0
**2**	0	0	0	0	0	0	0	0
**3**	0	0	0	0	1	0	1	0
**4**	0	0	0	0	0	0	0	0
**5**	0	0	0	0	0	0	0	0
PII	0/10 = 0		0/10 = 0		0/10 = 0.1		1/10 = 0.1	

For erythema: 0 = No erythema, 1 = very slight erythema, 2 = well-defined erythema. For edema: 0 = No edema, 1 = very slight edema. Irritation Index (PII): Negligible 0–0.4, Slight irritation 0.5–1.9, Moderate irritation 2.0–4.9, Severe irritation 5.0–8.0

### Textile application

**Fig. 10:**
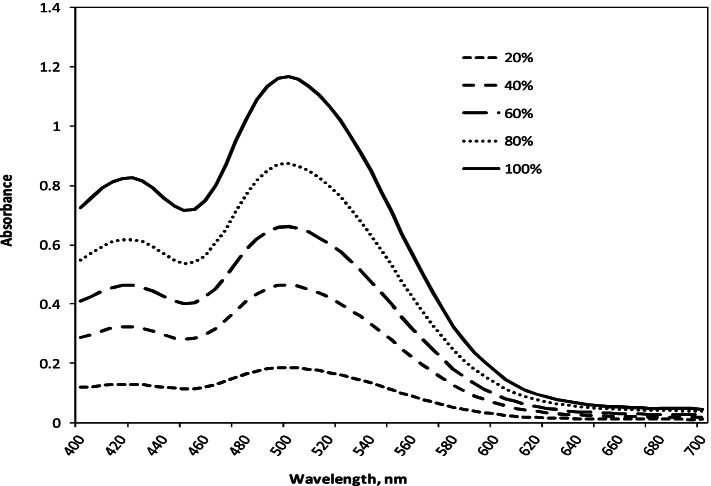
The absorption spectra of the produced pigment of *T. atroroseus* TRP-NRC water extract at various concentrations (20-100%) and their absorption maxima at *λ*_max_ 500 nm

**Fig. 11:**
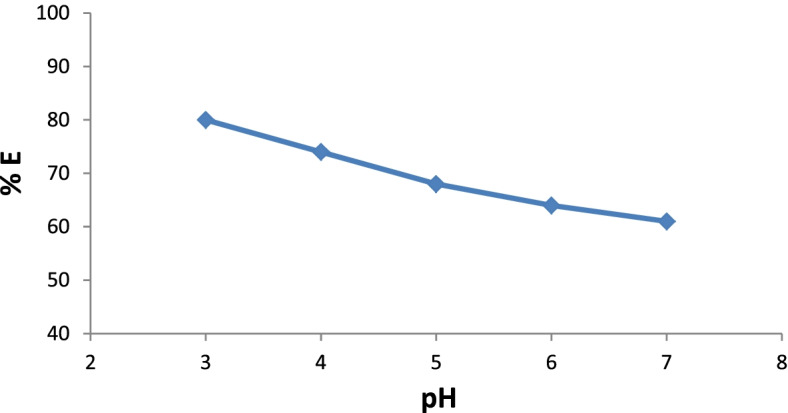
Effect of dyebath pH on the pigment exhaustion for the produced pigment of *T. atroroseus* TRP-NRC (100% conc.) on wool fabrics

**Fig. 12:**
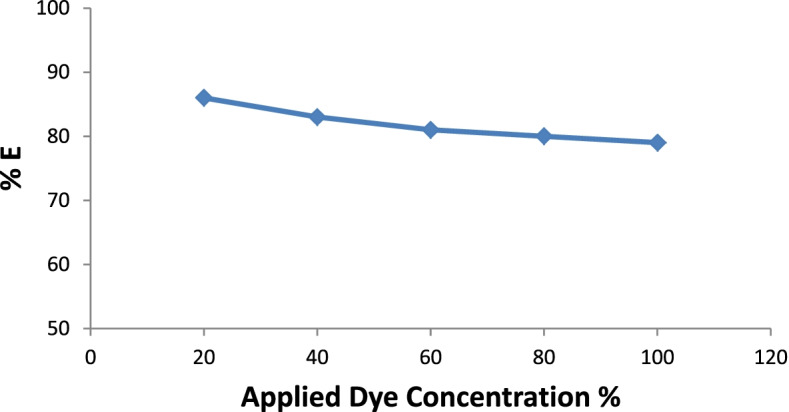
Effect of dye concentration on the pigment exhaustion for the produced pigment of *T. atroroseus* TRP-NRC on wool fabrics

**Fig. 13:**
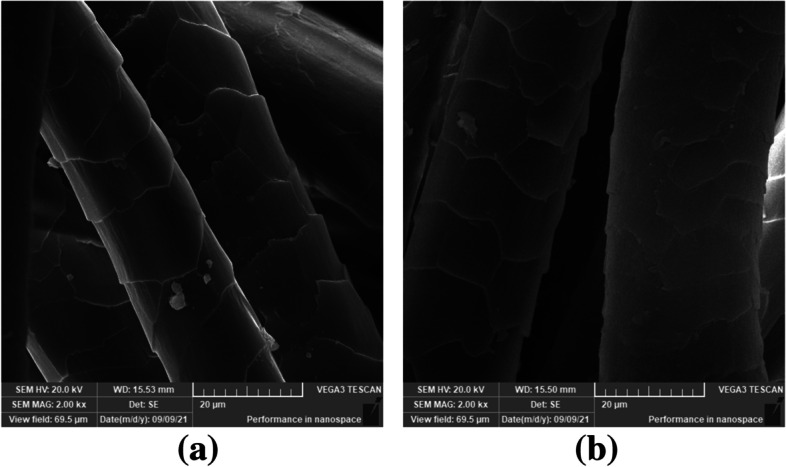
Scanning electron microscopy (SEM) images of the wool fabric: **a** Before dyeing; **b** after dyeing with 100% *T. atroroseus* pigment extract

Initially, the pigment was evaluated by measuring its maximum absorption in various aqueous solutions with a strength of 20, 40, 60, 80, and 100%, and the absorption spectra were obtained (Fig. 10). The absorption maximum became sharper and higher when the pigment concentration increased from 20% to 100% at a *λ*_max_ of 500 nm. The results showed the effect of the *p*H values of the pigment bath on the dyeing ability for wool fabrics using 100% strength (Fig. 11 & Table 5). Moreover, the extent of the pigment/fiber interaction was also assessed by determining pigment exhaustion and the K/S on wool at *p*H 3 using various concentrations (Fig. 12 & Table 6). Similarly, the results of color fastness to washing, perspiration, and light investigations were also confirmed on wool fabric (Table 7). The surface morphology of dyed wool fabric samples compared with the undyed sample was further examined by SEM (Fig. 13).

**Table 5:**
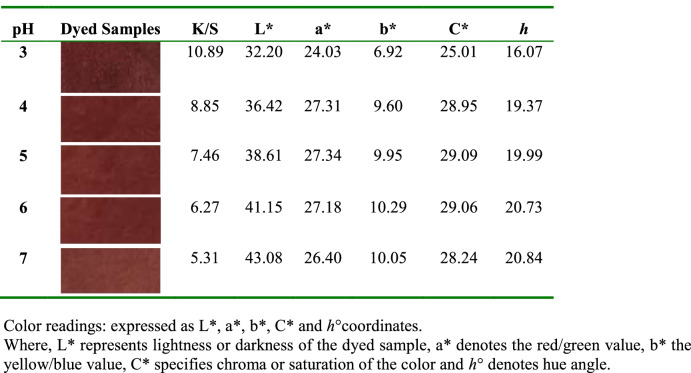
CIE Lab results of wool fabrics dyeing with the *T. atroroseus* TRP-NRC extracted pigment (100% conc.) at different *p*H

**Table 6:**
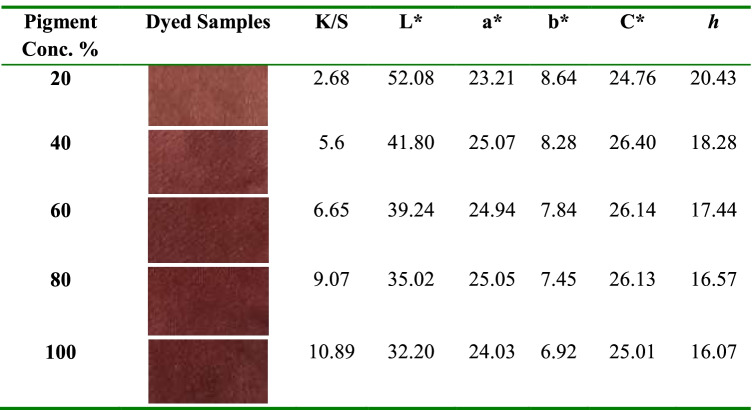
CIE Lab results of wool fabrics dyeing with the *T. atroroseus* TRP-NRC extracted pigment at different concentrations

**Table 7:** Color fastness properties of the dyed wool fabric using 20 and 80% pigment concentrations

Dyed Sample	Washing fastness			Perspiration fastness						Light
				Acidic			Alkaline			
	CC	Sc	Sw	CC	Sc	Sw	CC	Sc	Sw	
**20%**	4–5	4–5	4–5	4	4–5	4	4–5	4–5	4–5	3
**80%**	4–5	4–5	4	4	4–5	4	4–5	4–5	4	3

*CC* color change, *Sc* color staining on cotton, *Sw* color staining on wool

## Discussion

It is worth mentioning that many reports recommend *T. atroroseus* as an effective producer of the azaphilone biosynthetic families mitrorubrins and *Monascus* like without any mycotoxins. In the literature, ITS sequencing as a fungal barcoding gene and PCR analysis were used as the most efficient strategy for the molecular identification of *T. verruculosus* [26], some wood-inhabiting fungi [51], and some *Aspergillus* isolates [52], for example. Morphological characterization of the fungal isolate revealed its descriptive form in terms of the shape of the conidia, spores, and hyphae. Although morphological characteristics are useful for species description, they are limited by the infrequent macroscopic structures [53], other than cryptic species complexes that are present in many taxa [54]. Therefore, molecular tools were implemented to complement morphological ones. Because of the high polymorphism of the non-coding ITS region and sufficient taxonomic units, this region is employed to classify sequences at the fungal species level. The advantage of using ITS as a DNA barcoding lies in the enormous reference sequences present in the NCBI sequence databases, i.e., GenBank, EMBL, and others; thus, the ITS region has been proposed as the primary fungal barcoding and is routinely used for identification, systematics, and phylogenetics [55].

Regarding the maximization of the production for the target red pigment, all previous studies recommended SSF-fermentation as the optimum approach [56–61]. Many studies applied submerged cultures for the production of a fungal red pigment from *Penicillium purpurogenum* and *Talaromyces purpurogenus* [56–60]. Moreover, SSF-fermentation was evaluated for the production of red pigment by *Penicillium purpurogenum* GH [61]. In the current study, cultivation under liquid static conditions had many advantages, such as reduction of both production cost and contamination. This was the first report that used an economic YMP medium with 2% mannitol as a carbon source to maximize pigment production. This study disagreed with other studies that reported that glucose seemed to be the best carbon source for red pigment production by *T. purpureogenus* KKP and other *Talaromyces* spp. [11, 59, 60]. An additional two studies reported that soluble starch and d-xylose are favorable carbon sources for the maximization of red pigment production from *Paecilomyces sinclairii* and *Penicillium purpurogenum* GH2, respectively [56, 62]. In contrast, sucrose was reported as the best carbon source for mycelia growth; however, it reduces red pigment production. As the *p*H is one of the key factors affecting the production of pigments, it was investigated here in the context of red pigment production [56]. Other studies revealed that an acidic medium (*p*H 6) achieved a high red pigment yield from *Paecilomyces sinclairii* and *T. purpureogenus* KKP [56, 60, 63]. *T. amestolkiae* produced its best pigment yield when the initial *p*H medium was 7.0 [58]. The effects of *p*H and temperature on red pigment production were evaluated in a submerged culture of *Penicillium purpurogenum* GH2, where the highest yield was recorded at a *p*H of 5 [62]. Although static fermentation has a low cost, it is not common in the research conducted for pigment production. Most researchers use the submerged culture technique with 20% v/v of fermentation medium to fermentation vessel capacity [11, 60, 61]. Herein, red pigment production was maximized at a *p*H range of 2–4.5, with 50% fermentation medium. This is considered an important economic point when considering the production feasibility at pilot or industrial scales. As new promising trials, the use of γ-rays was considered a mutation-induction parameter for enhancing pigment production. Many studies examined the effect of different doses of γ-radiation (250–2000 Gy) on spore germination and the production and efficacy of penicillin from *Penicillium chrysogenum* [64–66]. It was found that the spore germination and penicillin yield were raised by 70.2% and 95.2%, respectively, at a dose of 200 Gy for 20 h compared with control cultures [64]. The γ-irradiation mutagenesis has led to an increase in the production of the anticancer drug paclitaxel by 1.22- and 1.24-fold in two mutants from *Aspergillus fumigatus* and *Alternaria tenuissima*, respectively, compared with their respective parent strains [65]. ISSRs have proven to be a powerful fingerprint technique because of the generation of a high level of polymorphism; therefore, they are useful for the study of closely related individuals with low polymorphism levels [67]. Various molecular markers, such as RAPD, RFLP, and AFLP, have been used by researchers for distinguishing fungal species. However, ISSR is not common in fungi; nevertheless, some researchers have used ISSR to distinguish specific varieties of mushrooms [68]. ISSR sequencing was employed to identify and distinguish a particular group of fungal pathogens that infect mango [67]. Moreover, a dose of 200 Gy duplicated the production of the prodigiosin drug as an antimicrobial, anticancer, and immunosuppressive red pigment [66]. In the present study, mutation was tried in a *Talaromyces* isolate using different doses of γ-radiation, to obtain maximum productivity of the red pigment. The ISSR-3 primer is considered the most efficient one regarding the total number of bands amplified, which may include several common (monomorphic) ones. However, the polymorphic bands produced by the primer relative to the total number formed by all primers represent the discriminatory power. Therefore, ISSR-3 has the highest discriminatory value, with 14 polymorphic bands including six non-unique polymorphic bands and eight unique polymorphic bands [69–72]. In the literature, the ISSR molecular markers were used effectively to evaluate the genetic microbial diversity and phylogenetic relationships [70], e.g., distinguishing among 40 *Aspergillus* isolates [71] and 59 rice blast fungus strains [72]. Polymorphic bands are powerful in the molecular differentiation between different individual species; PCR-ISSR generates patterns of banding that are unique to individual species, in addition to exhibiting or lacking unique band(s) that distinguish an individual from the remainder of the population [72]. Therefore, the discriminatory power of a primer is more important than its efficiency in determining its DNA fingerprint. These variations in ISSR banding patterns might be connected with structural rearrangements in DNA caused by the different types of DNA damage caused by gamma mutagenesis. These results showed that ISSR primers are a robust and informative maker and would be a better tool for genetic-diversity and phylogenetic studies. In the genetic distance and cluster analysis of ISSR-PCR, it was observed that ISSR-PCR provides more informative data for genetic diversity analysis. It can be concluded that random mutation, which affects the pigment-production pathway, resulted from the formation of free radicals and reactive oxygen species triggered by the effect of γ-irradiation on fungi. The mechanisms of mutation and increased pigment concentration may be explained by the increase in fungal membrane permeability and gene copy number, or upregulation of the expression of genes involved in pigment production [73].

The abundances of different ions in the LC/MS chromatograms are normally correlated with the relative stabilities of their own metabolites, as positive or negative forms. The majority of the metabolites of *Talaromyces* spp. were identified previously; in contrast, many of them were identified for the first time in *T. atroroseus* [7, 10–25, 74]. It is worth mentioning that a total of 80 peaks with reasonable relative concentrations were detected in both TIC chromatograms, containing 60 known and many unknown fungal metabolites. This depended on the chemotaxonomy, *R*_*t*_-values, and literature comparison with the corresponding exact masses of quasimolecular ions ([M+H]^+^, [M–H]^−^) and/or some induced adduct ions, e.g., [M+Na]^+^, [M+K]^+^, and [M+HCO_2_]^−^, editing more 23, 39 and 45 amu [10, 12, 18–25]. It is also of importance to pay attention to the isolation and identification of the unknown high-MW metabolites (> 700 amu) using different tools of analysis, where it is expected that they will contribute strongly to both the red color and biological effects of the pigment. Most of such metabolites were detected as negative ions, e.g., [M-H]^–^ at *m/z* 970.5933 (ID: 11); 869.9120, 916.8452, 933.0507 (ID: 30,31); 987.8199 (ID: 32); 918.9837, 931.9302 (ID: 35); 905.8402 (ID: 36); 989.1996 (ID: 37); 916.9109, 987.9391 (ID: 48,49); 869.9540, 988.0034 (ID: 50); 870.0154, 885.9044 (ID: 52); 916.0376 (ID: 54); 915.9235 (ID: 56); 929.9794 (ID: 80); and as [M+H]^+^ and [M+Na]^+^ at m/z 815.4283 and 837.5881 (ID: 16); 805.5860 (ID: 20); 885.8535 and 907.8815 (ID: 79). Finally, the findings confirmed that the new local *T. atroroseus* TRP-NRC isolate produces the known *Monascus-*pigment constituents [13–17], such as PP-R (12), azaphilone pigment (29), purpuride (31), PP-V (37), sophoraisoflavanone-C (40), PP-Y (46), ZG-1494a (48), monascin (50), and orange azaphilone pigment or sclerotiorin (59). For the first time, mitorubrin derivatives, i.e., mitorubrinolamine acetate, 3 (ID: 3); dihydro-PP-O (ID: 24); mitorobrinal 43 (ID: 25); and mitorubrinol 45 (ID: 26), were identified in *T. atroroseus* species in the current study. Moreover, these metabolites have been identified before in other *Talaromyces* species [74]. The structures of the metabolites are shown in Fig. 9.

The recorded range of the skin irritancy score (0.0–0.1) confirmed the negligible effect or non-irritancy of the target pigment and its safety for the human skin in the context of its application for textile dyeing (Table 4). According to the literature, no reports were available on the effect of the target pigment on the formation of skin erythema and edema. A previous study reported that fungal pigments (*Monascus* and *Monascus*-like pigments) are widely used in the cosmetic industry as sunscreens, sun lotions, sunblocks, face creams, anti-aging facials, and many others [75].

A significant effect of *p*H on the pigment exhaustion and K/S values of the dyed samples on the change was observed. This could be attributed to the correlation between the chemical structures and physical bonding of the constitutive metabolites within the treated wool fiber. It was of interest that the highest pigment uptake was noticed at *p*H 3, and then decreased with increasing *p*H value. This may be explained by the decreasing number of protonated amino groups on wool fibers, which decreases the ionic interaction [76]. The improved pigmenting observed at *p*H 3 and 100% strength was further confirmed by its lower L* values of the color developed on wool fabric. All dyeing processes showed positive a* and b* values, indicating that the appearance color of the treated samples seems to be in the same color space quadrant, even at a higher bath *p*H and lower pigment strengths of 20%, 40%, 60%, and 80%. The produced colors of the dyed samples closely matched red rather than yellow, referring to the higher values of a* than those of b*. Pigmented wool fabric also exhibited very good ratings of washing and perspiration fastness (Table 7). However, the ratings of the light fastness seem to be fair to good, which was in agreement with nearly all pigments.

The SEM images demonstrated the significant effect on the surface of wool samples when dyeing with 100% *T. atroroseus* pigment extract (Fig. 13). The sharp scales of wool were obliterated on the cuticle surface because of the uniform distribution and penetration of the dye molecules.

## Conclusions

It could be concluded that the identified *T. atroroseus* TRP-NRC is a promising isolate for red pigment production. The pigment production was effectively improved by γ-rays, and maximum production was recorded (27.36 g/L) by the mutant TRP-NRC 400 Gy at a dose of 400 Gy, which is estimated as a 30% higher pigment concentration under optimized conditions compared with the unexposed wild type. Our findings revealed that ISSR-PCR is the most powerful strategy for distinguishing between the wild-type *T. atroroseus* TRP-NRC strain and its mutants. LC/HRESI-MS analysis potentially revealed a high structural diversity of medium/high-molecular-weight metabolites, including 60 known and the most common active coloring metabolites reported in the fungal red pigments. Moreover, four mitorubrin derivatives, i.e., mitorubrinolamine acetate 3 (ID: 3), dihydro-PP-O (ID: 24), mitorobrinal 43 (ID: 25), and mitorubrinol 45 (ID: 26), were identified for the first time in *T. atroroseus*. This technique has led to the detection of the monoisotopic masses of a large number of high MWs metabolites that may be of new structures and effectively contributed to the color and other applications of the pigment. Accordingly, an extensive separation and identification study of such metabolites is currently being considered. Moreover, it is proven that the pigment is safe for the human skin and suitable for wool fabric dyeing. Because of the current findings pertaining to green, economic, and sustainable pigment production, the isolate reported here could be used in the future for other vital applications, e.g., food, cosmetics, or different pharmaceutical industries after carrying out complete and conventional studies of its safety and toxicity, which are currently being investigated.

## Supplementary Information

**Additional file 1: Figure S1.** Representative MS spectra for some metabolites identified using both ion modes from the total pigment of *T. atroroseus* TRP NRC water extract, corresponding to some peaks of different ID (see Table 3); Bold-underline numbers= serial numbers for identified metabolites.

## Data Availability

The datasets used and/or analyzed during the current study are available from the corresponding author on reasonable request.

## Abbreviations

UHPLC/HRESI-MS: Ultra-high-performance liquid chromatography/high-resolution electrospray ionization-mass spectrometry
PP-O, -V, -Y, -R: *Penicillium purpurogenum*-orange, -violet, -yellow, -red
ITS: Internal transcribed spacer
PCR: Polymerase chain reaction
ISSR: Inter-simple sequence repeat
MP: *Monascus* pigment
RAPD: Random amplified polymorphic DNA
AFLP: Amplified fragment length polymorphism
YMP: Yeast malt peptone agar
SEM: Scanning electron microscope
