# Supplementary figures and images for: A local Talaromyces atroroseus TRP-NRC isolate: isolation, genetic improvement, and biotechnological approach combined with LC/HRESI-MS characterization, skin safety, and wool fabric dyeing ability of the produced red pigment mixture
Source: J Genet Eng Biotechnol. 2022 Apr 22;20:62. doi: 10.1186/s43141-022-00335-2 (PMC9033925; doi:10.1186/s43141-022-00335-2)

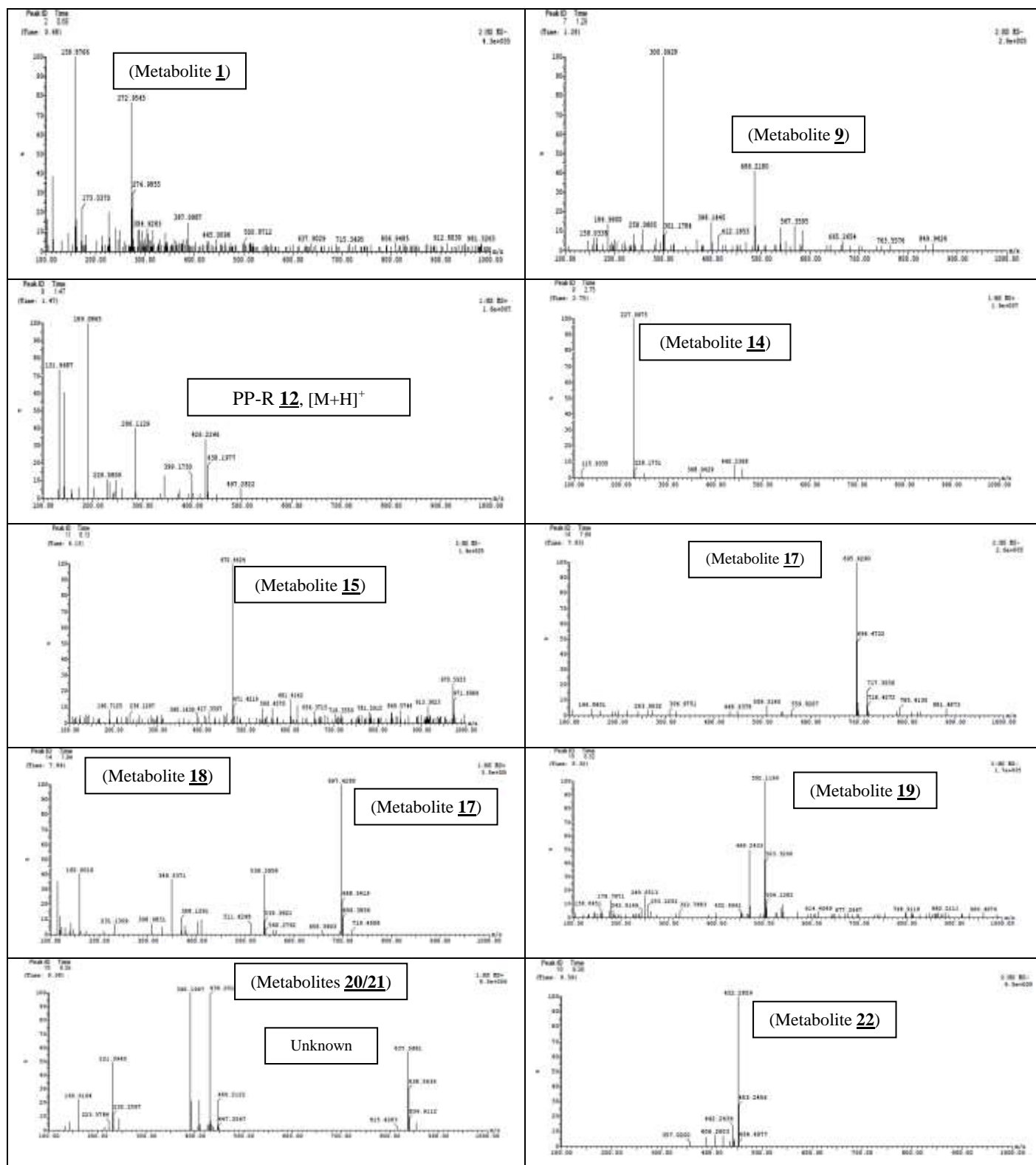

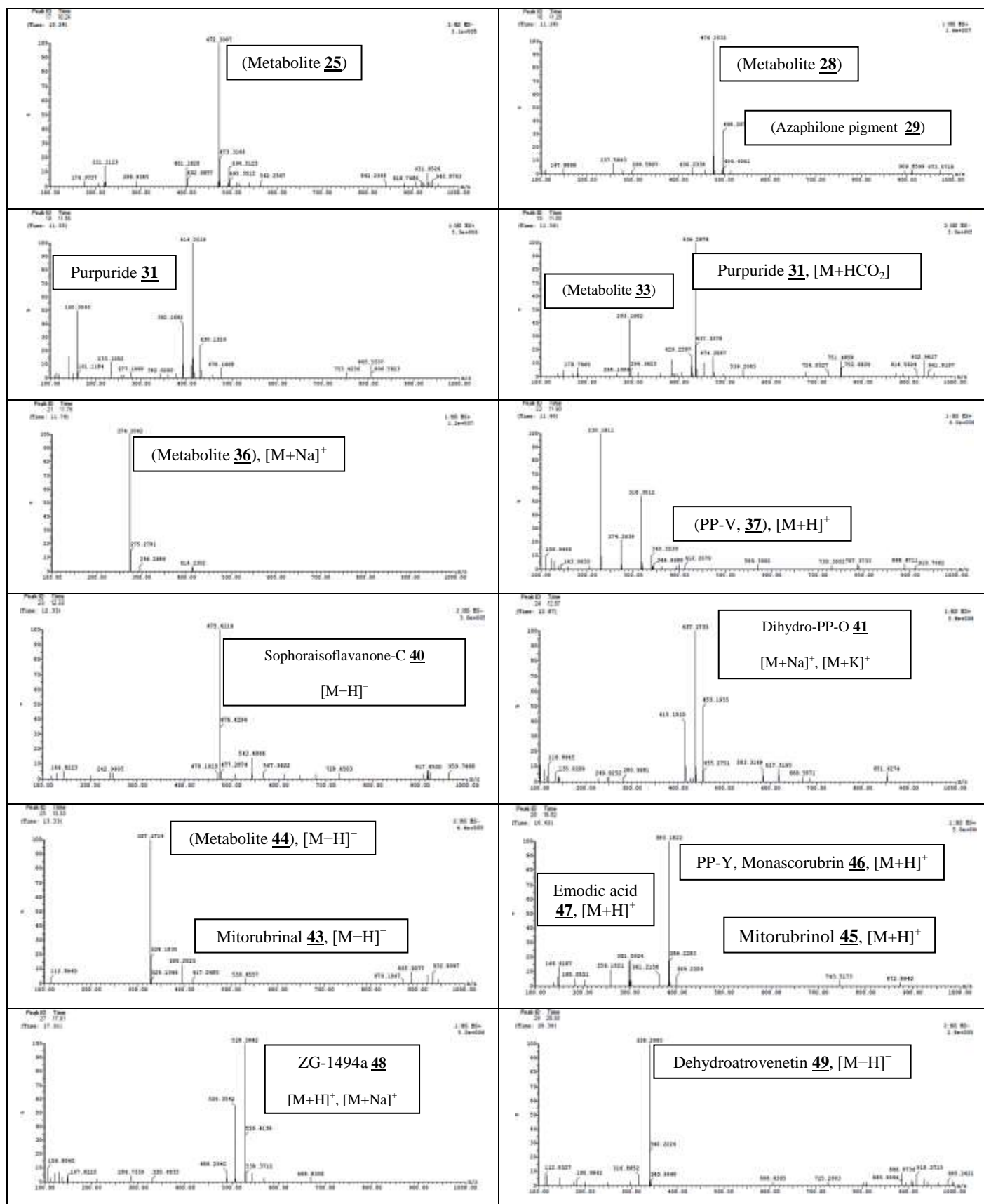

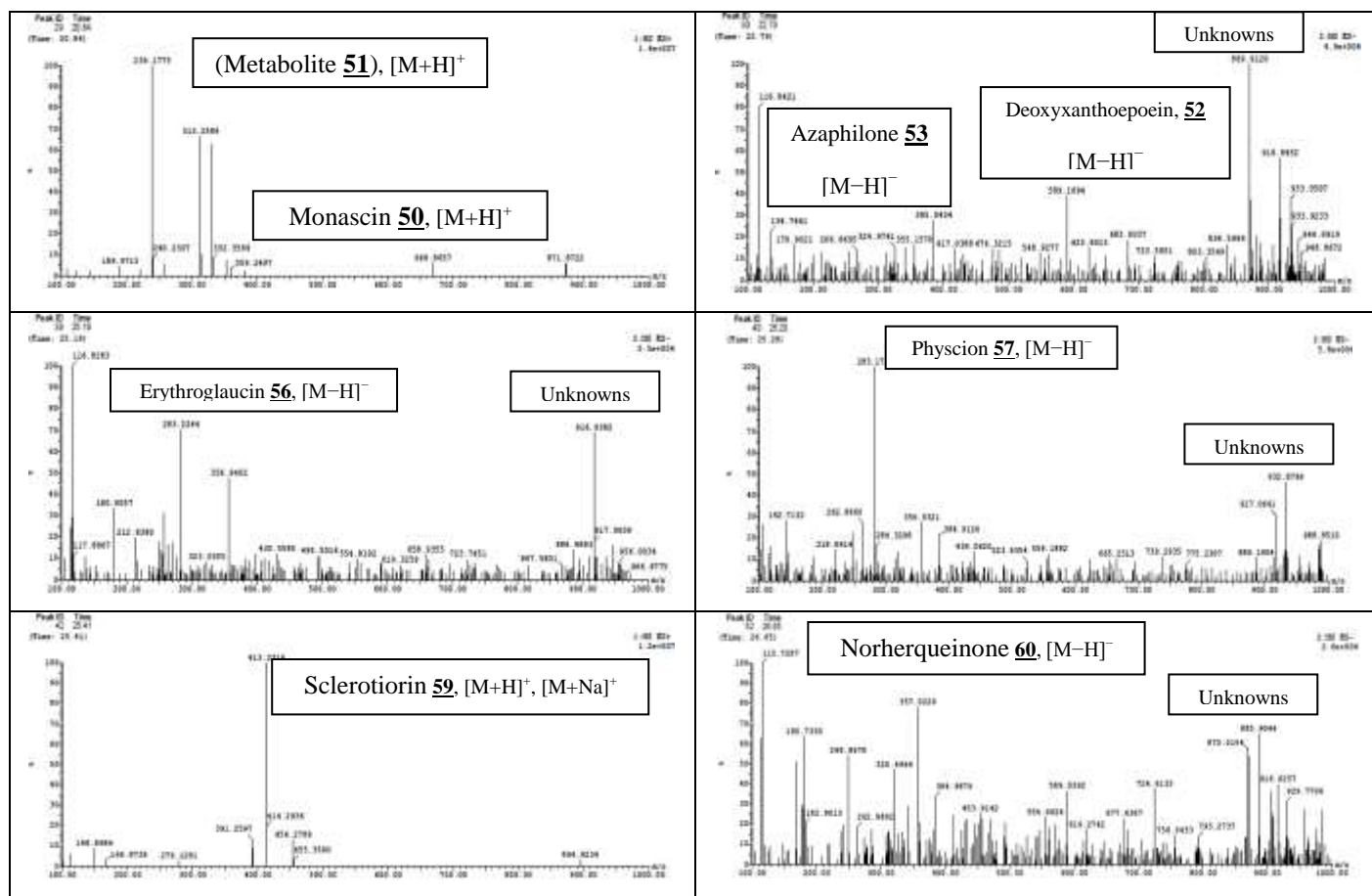

Supplement: Supplementary file 1 — Additional file 1: Figure S1. Representative MS spectra for some metabolites identified using both ion modes from the total pigment of T. atroroseus TRP NRC water extract, corresponding to some peaks of different ID (see Table 3); Bold-underline numbers= serial numbers for identified metabolites. [file 43141_2022_335_MOESM1_ESM.pdf]
